# Tantalum recovery from alkaline leachates based on magnesium hexatantalate intermediate precipitate†

**DOI:** 10.1039/d5ra02721g

**Published:** 2025-06-13

**Authors:** Mbolantenaina Rakotomalala Robinson, Rana Choumane, Domitille Giaume, Grégory Lefèvre

**Affiliations:** a Chimie ParisTech, PSL Research University, CNRS, Institut de Recherche de Chimie Paris (IRCP) F-75005 Paris France gregory.lefevre@chimieparistech.psl.eu

## Abstract

A new approach for the recovery of tantalum (Ta(v)) from alkaline solutions is presented, with the expected environmental benefits. This method is based on magnesium-induced precipitation to extract tantalum present as polyoxotantalates (POTas) from alkaline leachates. We obtained a tantalum precipitation yield of over 99% by introducing a Mg/Ta_6_ ratio of ≥4 into the solution. We show that Ta(v) ions, as hexatantalate species, precipitate in the presence of magnesium ions to form Mg_4_Ta_6_O_19_·*x*H_2_O crystalline phases with two different structures, with *x* = 16.1 or 24.4. The magnesium tantalate precipitate is then dissolved in ethylenediaminetetraacetic acid (EDTA) to release Mg(ii) in the form of an Mg(EDTA)^2−^ complex. The final product, amorphous hydrated tantalum pentoxide (Ta_2_O_5_·nH_2_O), is obtained by neutralizing the Mg-EDTA-POTa solution to pH 7. After thorough washing with dilute acid and ultrapure water and calcination, tantalum pentoxide is obtained.

## Abstract 2

Extraction of soluble Ta(v) *via* precipitation with Mg(ii) and recovery as Ta_2_O_5_ solid.

## Graphical abstract

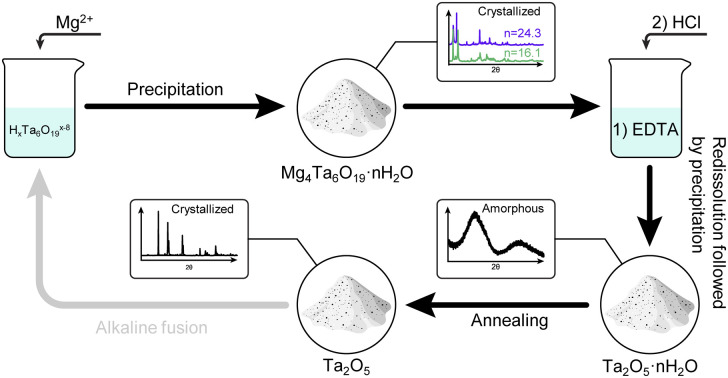

## Introduction

The chemical element tantalum (Ta) belongs to the group of refractory metals. Its extraordinary properties, such as chemical inertness and high melting point, make it valuable in various fields of technology.^1^ Although the electronics industry consumes almost half of the tantalum extracted from ores for the manufacture of capacitors, this element also has applications in aerospace, medical equipment, and industrial equipment sectors.^2,3^ In the European Union, tantalum is on the list of the 34 critical and strategic raw materials because of the potential risks associated with its supply.^4^ Its current production is limited to a few countries, but the global geopolitical situation can change at any time. The potential interruption of its supply chain can have catastrophic consequences on the activities that depend on it. Therefore, it is necessary to exploit all available sources of its supply. In this context, research is focusing on the recycling of waste from electrical and electronic equipment (WEEE), in particular tantalum capacitors, which are still under-recycled but represent a significant reserve of tantalum. Compared with natural ores, tantalum capacitor waste has the advantage of high tantalum content and the absence of niobium; however, other impurities may be present, such as nickel, iron and silicon.^5,6^ Based on existing processes for extracting and purifying tantalum from ores, several hydrometallurgical processes may be suitable for its recovery from e-waste. The most common process involves the use of concentrated hydrofluoric acid to dissolve tantalum, which is usually associated with niobium in ores. The resulting leach solution is subjected to a solvent extraction process to separate Ta and Nb, as well as other elements present.^7,8^ This method has been criticized on safety and environmental grounds owing to the use of hydrofluoric acid, which is an extremely toxic and corrosive agent. The use of alkaline melting at moderate temperatures compared with pyrometallurgical processes to form soluble tantalum compounds has attracted much interest in recent years.^9–11^ In its metallic or oxidized form (Ta_2_O_5_), tantalum is insoluble in water, but alkaline fusion generates highly negatively charged soluble polyoxotantalates (POTas) H_*x*_Ta_6_O_19_^*x*−8^ (0 ≤ *x* ≤ 3 or 4) in an alkaline environment. The maximum number of protonations is still a matter of debate as no consensus has yet been reached.^12,13^ Consequently, hydrometallurgical processes aimed at recovering Ta *via* alkaline fusion must consider the treatment of these polyoxotantalate ions in solutions. Precipitation of tantalum in the form of hydrated Ta_2_O_5_ from basic leachates can be achieved by acidifying the solution, but this method has several drawbacks,^14^ and precipitation of Ta(v) without neutralization of the leaching solution is an option worth exploring. To extract POTa from alkaline leachate, we have previously attempted to take advantage of the ion exchange properties of Layered Double Hydroxides (LDHs).^15^ We used Mg–Fe–CO_3_ LDH to replace the carbonate ions in the material with POTa. However, the results were not satisfactory, as only 14% of the anions of the material were exchanged owing to the high affinity between LDH and carbonates. After calcining the Mg–Fe–CO_3_ LDH at 400 °C to remove carbonates in the form of CO_2_, we expected to extract more POTa when reconstructing the LDH in the solution. This was indeed the case, but we concluded that the mechanism involved the formation of a magnesium-POTa precipitate rather than the adsorption of POTa into the interlayer space of the LDH. The hypothesis of precipitate formation was validated by the appearance of a white precipitate when highly soluble Mg salts (MgO and MgCl_2_) were dissolved in the POTa solution. A similar study^16^ demonstrated the formation of a precipitate when a solution containing Ca^2+^ ions of different salts was mixed with a sodium or potassium hexatantalate solution, as well as with a solution of sodium hexaniobate, or a mixed solution of hexaniobate and hexatantalate. The precipitation yields of niobium and tantalum are very high, close to 100%. The use of guanidine as a precipitating agent also resulted in a high tantalum precipitation yield (>99%). Guanidine is an amine that forms a highly stable cation (C(NH_2_)_3_^+^) in an aqueous solution and forms a white precipitate by electrostatic interaction with POTa ions.^14,17^ The ability to precipitate tantalum using either an alkaline earth metal or an amine opens up a new way of recovering tantalum from alkaline leachates. The precipitation products should undergo additional processing to obtain the target product, Ta_2_O_5_, with a purity compatible with electronic device applications. Guanidine-Ta precipitates are calcined at high temperatures (900 °C) to decompose organic matter while simultaneously producing crystallized Ta_2_O_5_. Although this approach is effective, a potential limit of the process lies in its significant energy consumption required to reach and maintain the high temperature and gaseous emanation that may cause health and environmental risks. For Ca/Mg–Ta precipitates, to the best of our knowledge, there have been no studies in the literature that mention their use in a tantalum recovery process to generate Ta_2_O_5_. Calcination does not remove calcium or magnesium from the system, so the strategy to be adopted for the treatment of Ca/Mg–Ta precipitates should favour aqueous solution chemistry.

This study aims to deepen the understanding of the physico–chemical interactions governing the formation and dissolution of polyoxotantalate precipitates in alkaline media, as well as their conversion into high-purity tantalum pentoxide. In this context, we focus more specifically on the precipitation of magnesium hexatantalate species at basic pH and the mechanisms enabling the regeneration of tantalum pentoxide from this intermediate. To achieve this, we investigate the use of EDTA, a chelating agent that can selectively complex magnesium, thereby promoting the dissolution of the precipitate. This dissolution process, driven by the formation of stable and soluble Mg-EDTA complexes, shifts the equilibrium towards the release of tantalum into the solution. Finally, controlled acidification enables the selective precipitation of tantalum pentoxide, while magnesium remains in a solution in its complex form.

## Materials and methods

### Reagents

Tantalum pentoxide (Ta_2_O_5_, Thermo Fisher Scientific), sodium hydroxide (NaOH, VWR), magnesium chloride hexahydrate (MgCl_2_·6H_2_O, R.P. Normapur), nitric acid 65% (HNO_3_, VWR), hydrochloric acid 37% (HCl, Fisher Scientific), hydrogen peroxide 30% (H_2_O_2_, VWR) and ethylenediaminetetraacetic acid (EDTA, Sigma Aldrich) were of analytical grade and used as received without further purification. Ultrapure water (18.2 MΩ cm^−1^ resistivity) was used for all experiments.

The synthesis of Na_8_Ta_6_O_19_ · 24.5H_2_O (NaPOTa) was carried out by alkali fusion of Ta_2_O_5_ in NaOH, in accordance with the procedures from literature.^18^ Briefly, Ta_2_O_5_ (5.7 mmol) was melted with NaOH (0.11 mol) in a Ni crucible at 400 °C for 5 h. The resulting solid was mixed with cold water (30 mL) and then washed 3 times with cold water (40 mL each time). The filtrated solid was dried in a vacuum. The resulting white powder was dissolved in 80 mL of water and then heated for 2 h at 85 °C. After filtration, the filtrate was placed in a refrigerator for one day. A white precipitate was formed and dried in a vacuum. The product was identified by XRD powder to confirm its structure (ICDD PDF 00-024-0948 file).

Stock solutions of Ta and Mg were prepared by dissolving NaPOTa and MgCl_2_, respectively, in ultrapure water. The concentrations were then verified using ICP-OES.

### Batch precipitation

Precipitation experiments were carried out by mixing 50 mL of NaPOTa solution (1000 ppm as Ta) with the required volume of MgCl_2_ solution (0.4 mol L^−1^) in an Erlenmeyer flask. The exact volumes are shown in Table ESI1.† The mixture was then stirred for 24 h using a magnetic stirrer. The initial pH of the solutions was not adjusted but remained at their natural values after dissolving the corresponding solids. For the NaPOTa solution, the pH was approximately 11, and for the MgCl_2_ solution, it was around 6. The experiments at 20 °C were conducted in a climate-controlled room, while those at 40 °C were carried out on a magnetic stirrer equipped with a temperature controller. The suspensions were then vacuum filtered through a 0.65 μm pore diameter filter. The equilibrium concentrations in the clear filtrates were determined using ICP-OES. The precipitates were washed three times with ultrapure water and dried under vacuum for 24 hours before analysis.

### Magnesium hexatantalate dissolution and tantalum oxy-hydroxide precipitation

Magnesium tantalate precipitates were dissolved in several propylene flasks, each containing 50 mL of EDTA solution with a concentration ranging from 0.31 to 2.50 mmol L^−1^, all adjusted to pH 11 with KOH 10 M. Solids were added in excess to achieve a saturated state under experimental conditions. The flasks were placed on a back-and-forth shaker for 24 hours. A fraction of the liquid was then collected and filtered through a 0.65 μm pore size filter. A maximum precipitation yield of 100% was found, indicating that the particles were larger than this pore size. The clear liquid was analyzed by ICP-OES. This first series of experiments enabled us to determine the amount of EDTA required to dissolve a given quantity of magnesium hexatantalate.

To prepare tantalum pentoxide, magnesium hexatantalate was dissolved in an EDTA solution at a concentration of 2.50 mmol L^−1^ in an Erlenmeyer flask. The volume used corresponds to a 10% excess of EDTA over the magnesium contained in the magnesium hexatantalate precipitate. The mixture was continuously stirred with a magnetic stirrer until the solid was completely dissolved. When the suspension became clear, it was acidified by adding concentrated nitric acid dropwise to pH 7, in the pH range of the stability of tantalum oxide. A white precipitate was formed when the acid met the solution. Stirring was maintained for 30 minutes. The resulting precipitate was recovered by vacuum filtration through a 0.65 μm pore size filter and then washed 3 times with an HCl solution of pH 3 and 3 times with ultrapure water. The final product was vacuum dried for 24 hours. Each sample is the result of an experiment carried out under specific conditions.

### Analytical techniques

The solution concentrations of the elements of interest were determined by inductively coupled plasma with optical emission spectroscopy or ICP-OES (iCAP 6000, Thermo Scientific). Powder X-ray diffraction patterns were recorded from 2*θ* = 5 to 60° in the Bragg–Brentano geometry using a D8 Endeavour diffractometer from Bruker equipped with a cobalt anode. The ATR-FTIR spectra were collected using a Thermo Scientific Nicolet 6700 FT-IR instrument equipped with a dry air filter and a Mercury Cadmium Telluride (MCT) detector. The spectral resolution was set at 4 cm^−1^. Furthermore, the spectra obtained were an average of 256 scans. Energy-dispersive X-ray (EDX) analysis was carried out using a Zeiss Supra 35 Leo scanning electron microscope equipped with a Bruker XFlash 6160 energy-dispersive X-ray spectroscopy microanalyzer. Thermogravimetric analyses (TGAs) were conducted using STA 6000 (PerkinElmer). The sample was heated at a rate of 5 °C min^−1^ from 30 to 800 °C under a nitrogen flow of 20 mL min^−1^.

## Results and discussion

### Hexatantalate ion precipitation in the presence of magnesium

**Fig. 1:**
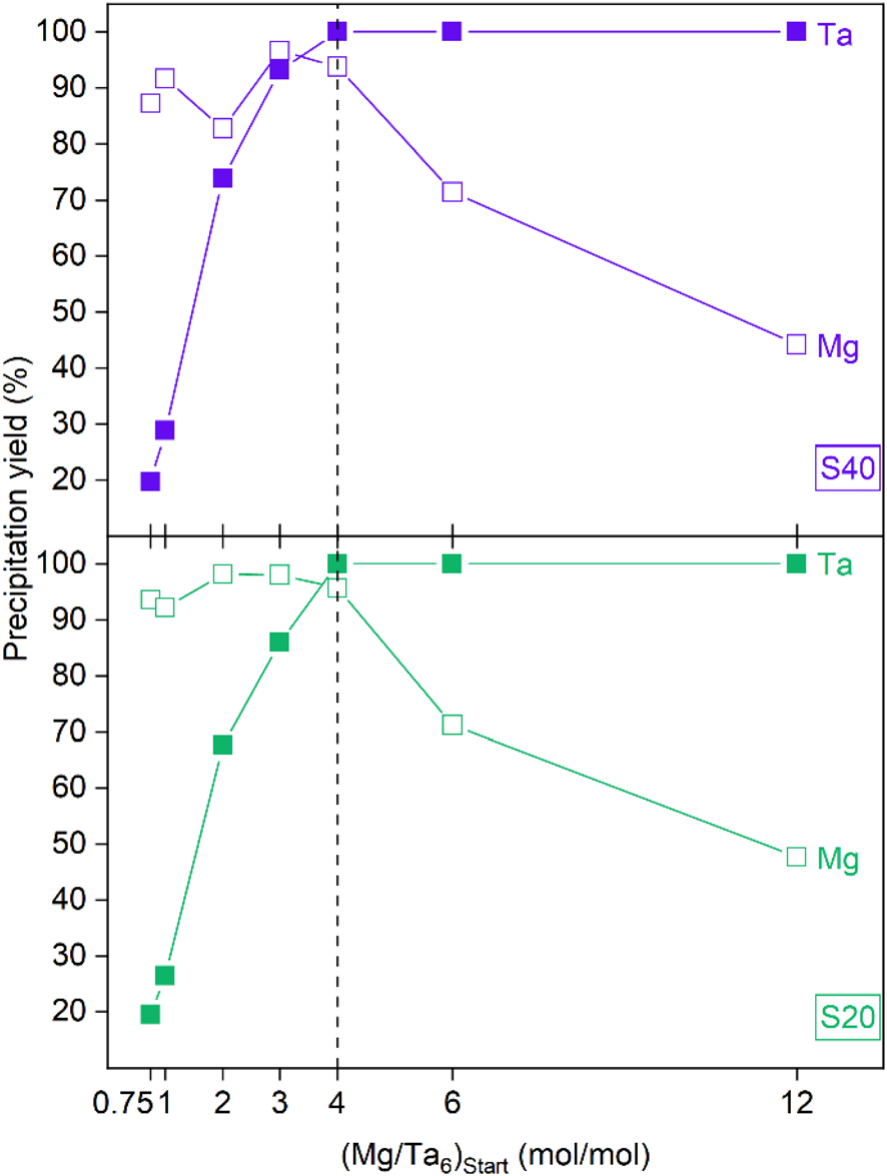
Precipitation yield of tantalum (Ta) and magnesium (Mg) after 24 h with the addition of MgCl_2_ solution to a sodium hexatantalate solution at 20 °C (S20 series) and 40 °C (S40 series). Initial tantalum concentration [Ta]_i_ = 5.53 mmol L^−1^; initial pH (pH_i_) = 11.

The precipitation of tantalum in an alkaline medium by magnesium was examined at two temperatures (20 °C and 40 °C) chosen to capture potential variations around typical laboratory or plant conditions. Fig. 1 illustrates the precipitation yield of hexatantalate (Ta_6_) by adding a magnesium chloride solution to a sodium hexatantalate solution at a pH of around 11. The expression of the ratio, Mg per 6 atoms of tantalum, is derived from the fact that tantalum exists in the hexatantalate form Ta_6_O_19_^8−^ in the studied solids. As the Mg/Ta_6_ molar ratio in the initial mixture increases, the precipitation yield of tantalum increases. Notably, the tantalum precipitation yield reaches its maximum, exceeding 99%, when the Mg/Ta_6_ ratio equals or exceeds 4. This suggests that a Mg/Ta_6_ ratio of 4 can subsequently be adopted to achieve the optimum tantalum precipitation yield in alkaline media, thereby avoiding the use of excess reagent (MgCl_2_). The total precipitation of tantalum at a ratio of 4 Mg to Ta_6_ would also imply the formation of a sparingly soluble precipitate of magnesium hexatantalate with composition Mg_4_Ta_6_O_19_. It could be assumed that 4 Mg^2+^ ions replace 8 Na^+^ ions to balance the charge of Ta_6_O_19_^8−^. The stoichiometry of the solid Mg_4_Ta_6_O_19_ ensures that the magnesium precipitation yield is high and remains relatively stable when Mg/Ta_6_ < 4 (excess of Ta relative to Mg). In the case of an excess of magnesium in the solution (Mg/Ta_6_ > 4), the precipitation yield of magnesium decreases owing to the excess magnesium not being incorporated into the compound. To confirm the elemental composition of the solid precipitates, they were analysed by ICP-OES after dissolution with H_2_O_2_ 30% and dilution in an HNO_3_/H_2_O_2_ mixture (Table 1). When exploring the influence of temperature, it was obvious that the variation from 20 °C to 40 °C insignificantly affected the composition of the tantalum precipitate. This implies that, under the conditions investigated, temperature variation does not change the precipitation yield.

**Table 1:** Ratios between Mg and Ta in the precipitate after the addition of MgCl_2_ solution to a sodium hexatantalate solution at 20 °C and 40 °C and 24 h of stirring and pH measured at room temperature. Initial tantalum concentration [Ta]_i_ = 5.53 mmol L^−1^

(Mg/Ta_6_)_start_	(Mg/Ta_6_)_solid_		Final pH	
	20 °C	40 °C	20 °C	40 °C
0.75	3.9	4.1	10.2	9.2
1	3.9	4.1	10.0	9.3
2	4.0	4.0	9.8	9.3
3	3.9	3.9	9.2	8.7
4	4.1	4.1	7.5	7.9
6	4.2	4.2	7.4	7.6
12	4.2	4.3	7.4	7.9
Average	4.0 ± 0.2	4.1 ± 0.1		

### Characterization of the Mg-POTas precipitates

Table 1 displays the results of the ICP-OES analysis on the solutions obtained following the dissolution of the solids.

Based on the Mg/Ta_6_ molar ratio values in solids illustrated in Tables 1, it appears that regardless of the initial Mg/Ta_6_ in solution and temperature, the resulting solid precipitates exhibit consistent Mg/Ta_6_ close to 4. This observation suggests that the precipitate consists of fixed stoichiometry.

These results are in contrast to what Deblonde *et al.* obtained when studying the precipitation of niobium by calcium from a solution of potassium hexaniobate.^16^ As Nb(v) and Ta(v) generally have similar chemistry, they are expected to exhibit similar behaviour. The amorphous precipitates obtained by Deblonde *et al.* comprised a mixture of K–Ca–Nb with a significant amount of potassium (K/Nb_6_ = 3 and Ca/Nb_6_ = 2.2). They mentioned the formation of ion pairs between K^+^ and the hexaniobate ion, leading to the co-precipitation of the latter in the presence of Ca^2+^. In their study, in NaCl medium, more calcium was required to precipitate niobium, which they justified by the fact that the ion-pairing effect between Na^+^ and the hexaniobate ion was less important. In our case, Mg^2+^ fully contributes to balancing the charge of Ta_6_O_19_^8−^, indicating that the incorporation of Na^+^ into the solid structure is unfavourable.

**Fig. 2:**
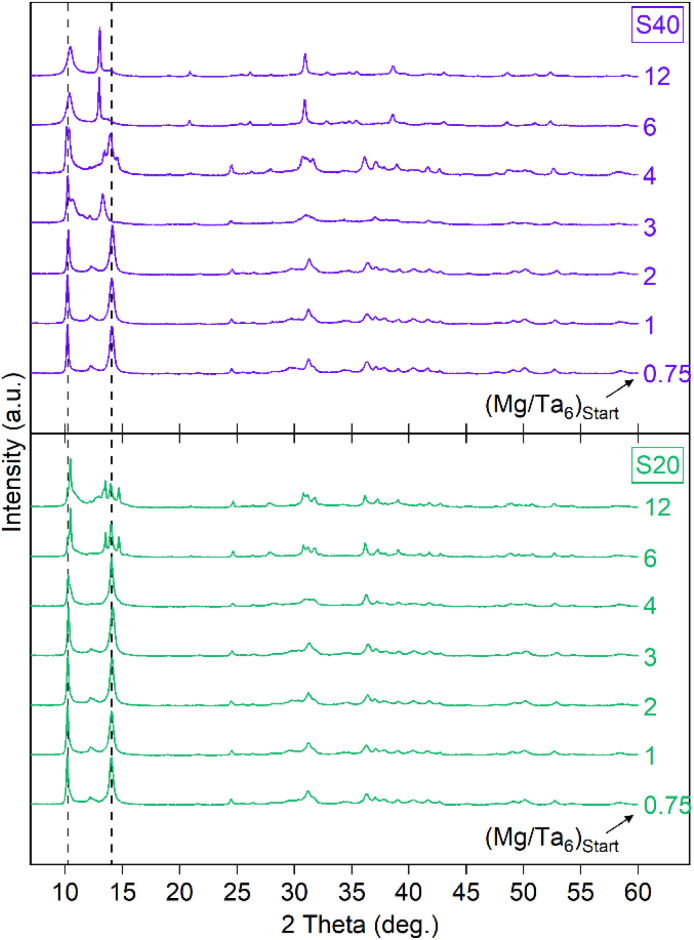
X-ray diffractograms of precipitates obtained at 20 °C and 40 °C for various initial Mg/Ta_6_ ratios.

We performed PXRD analyses on the solids precipitated between Mg^2+^ and hexatantalate to provide information on the structural properties of the materials formed during precipitation (Fig. 2).

Unlike the amorphous phases reported by Deblonde *et al.* when precipitating Ca^2+^ and K^+^ with hexaniobate,^16^ our materials exhibit distinct and sharp diffraction peaks, indicating a well-ordered crystalline structure. However, these reflections do not match any known phases in the available databases (ICDD and others), suggesting that these are new, previously unreported magnesium hexatantalate phases. Consequently, a standard phase identification or Rietveld refinement could not be performed. Future work will focus on growing crystals of sufficient size for single-crystal X-ray diffraction, which will allow us to fully elucidate the structure of these magnesium hexatantalate phases.

Although most diffractograms obtained show a similarity characterized by peaks at 10.3° and 14.1°, variations are observed in some diffractograms, such as those for solids precipitated at 20 °C with initial Mg/Ta_6_ ratios of 6 and 12 as well as those for solids precipitated at 40 °C with initial Mg/Ta_6_ ratios of 3, 6 and 12 (called S40-3, −6 and −12 hereinafter, respectively). It would indicate that, although possessing a similar chemical composition, the precipitated solid may exhibit differences in terms of crystalline structure owing to different polytypes (as reported for other related compounds, such as Na_8_Ta_6_O_19_ · 26H_2_O^19^) or owing to a different number of structural water molecules.

**Fig. 3:**
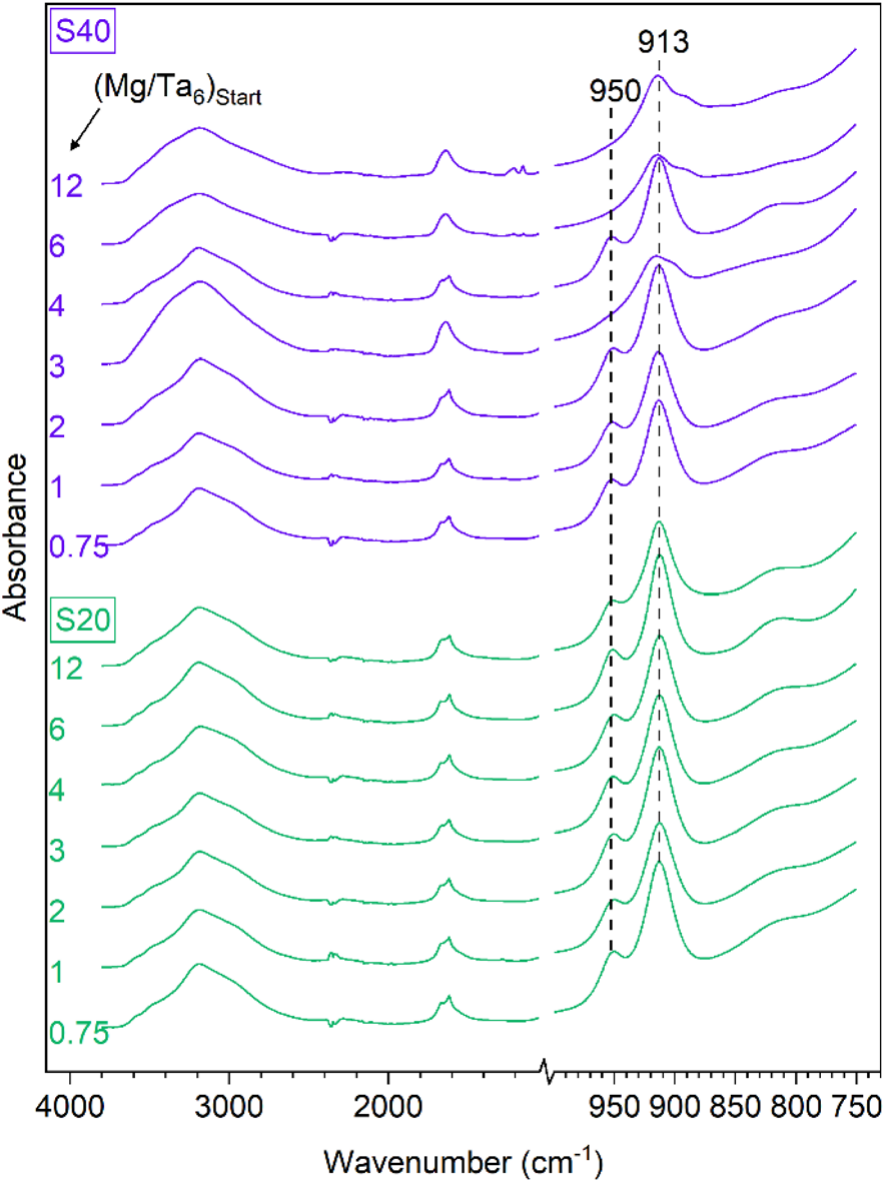
ATR-FTIR spectra of precipitates obtained at 20 °C and 40 °C for different initial Mg/Ta_6_ ratios.

To further investigate the nature of the compound(s), we performed FTIR analyses on the solids obtained at 20 °C and 40 °C. As seen in Fig. 3, characteristic vibration bands appear in the 850–1000 cm^−1^ region, consistent with some polyoxometalates.^20,21^ Two peaks are present in most samples at 913 and 950 cm^−1^, except for the precipitates S40-3, −6 and −12, mirroring the differences observed in their XRD patterns. The broad band around 3200 cm^−1^ and the peak at 1640 cm^−1^ can be attributed to the stretching and bending vibrations of water, consistently observed in hydrated compounds. Even if these bands are present for all samples, they are less sharp for the precipitates S40-3, −6 and −12, which would be consistent with a different water environment and/or content in these solids. To confirm this assumption, thermogravimetric analysis was used to determine the amount of water in each sample. The results are presented in Table S1 (ESI).† An average of 16.1 water molecules by Mg_4_Ta_6_O_19_ unit has been obtained, except for S40-6 and S40-12 where an average of 24.3 has been obtained, and S40-3 with an intermediate value of 19.0. These results confirm that two compounds corresponding to Mg_4_Ta_6_O_19_ · 16.1H_2_O and Mg_4_Ta_6_O_19_ · 24.3H_2_O would be consistent with these results. The origin of the difference in the hydration of the precipitates would not be linked with the initial Mg/Ta_6_ ratio, but would rather depend on different drying protocols. Further investigation on their structure is in progress but is outside the scope of this article and the hydration state of the compound has no impact on the following step of the process described below.

### EDTA leaching of MgPOTa precipitates in basic medium

**Fig. 4:**
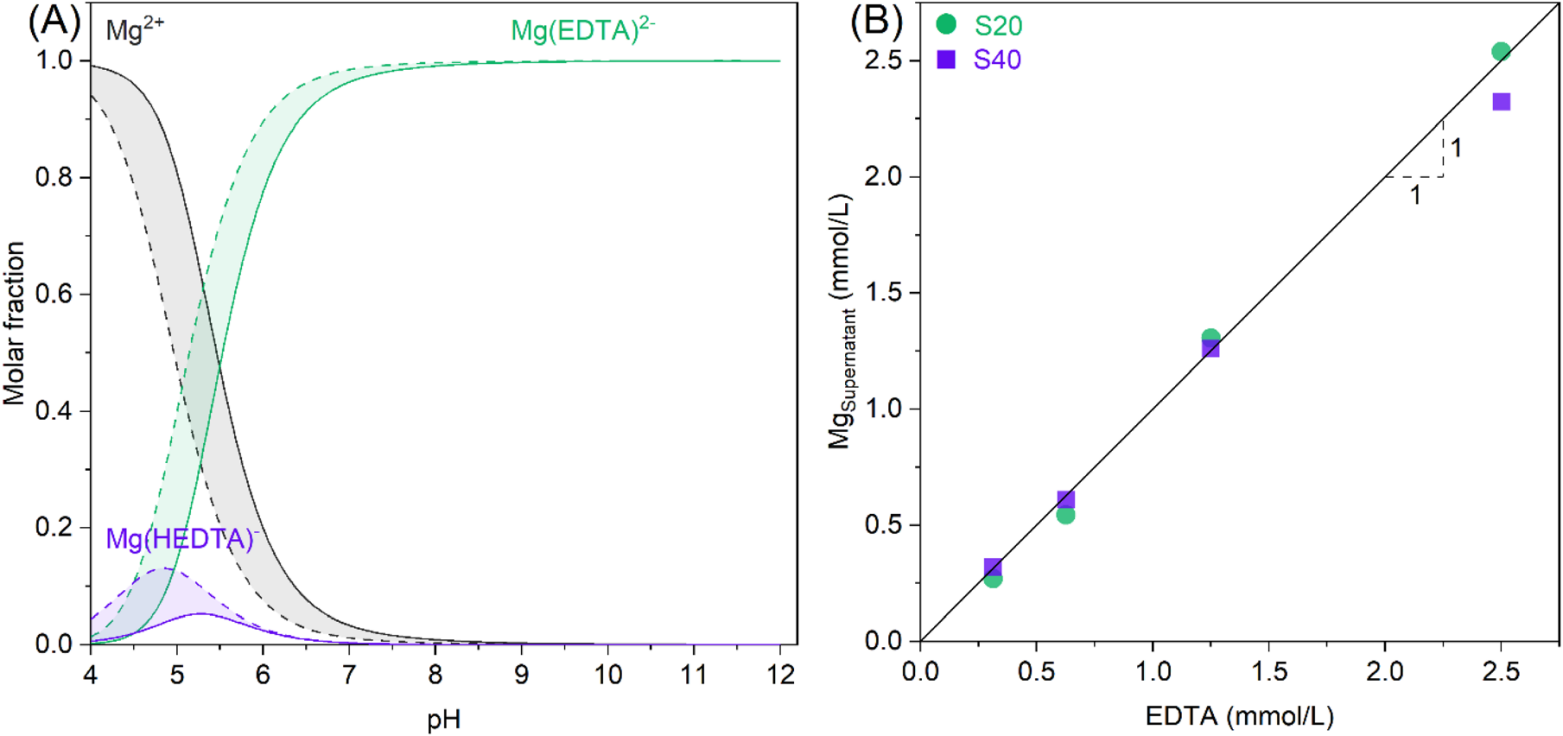
(A) Distribution diagram of magnesium species as a function of pH calculated using MEDUSA fed by the HYDRA database (Puigdomenech, 2004). The solid lines correspond to an equimolar mixture of Mg and EDTA of 0.31 mmol L^−1^, and the dashed lines correspond to an equimolar mixture of Mg and EDTA of 2.50 mmol L^−1^. (B) Effect of EDTA concentration on the equilibrium concentration of Mg after adding an excess of magnesium hexatantalate in EDTA solutions with varying concentrations at pH 11.

MgPOTa precipitates cannot be used directly from a Ta metal production perspective. In industrial practice, Ta_2_O_5_ is required as a starting material. Mg precipitation is only helpful for precipitating tantalum and forming Ta_2_O_5_; it is then necessary to dissolve MgPOTa precipitates. It is known that the solubility of a solid increases when a complexing agent for at least one of its constituents is present. In our case, we used EDTA, which can form a 1 : 1 complex (Mg(EDTA)^2−^) with magnesium from a mildly acidic pH according to thermodynamic calculations (see Fig. 4A). Another approach should be to consider the peroxylation of the hexatantalate species using H_2_O_2_,^22^ similar to the method used to stabilize Ta(v) in an acidic solution for ICP-OES measurements, but preliminary tests have shown stability issues of H_2_O_2_ in the medium, which decomposes quite rapidly with the release of O_2_.

Only precipitates obtained at 20 and 40 °C with an initial Mg/Ta_6_ ratio equal to 4 are considered in the subsequent experiments. Fig. 4B shows the equilibrium concentrations of magnesium in the leachate during the dissolution of precipitates obtained at 20 and 40 °C by EDTA with increasing concentrations between 0.3 and 2.5 mmol L^−1^ at pH 11. One can observe that the equilibrium concentration of Mg in the leachate increases with the concentration of EDTA used. Additionally, a linear relationship can be established between the concentrations of Mg and EDTA, as evidenced by the proximity of the data points to the diagonal line with a slope of 1. These results confirm the formation of a 1 : 1 Mg(EDTA)^2-^ complex during the dissolution of MgPOTa precipitates, subsequently providing an initial estimate of the quantity of EDTA required. To ensure complete dissolution of the solid, an excess of 10% EDTA relative to the stoichiometric quantity is employed.

### Recovery of Ta_2_O_5_ by acid precipitation

**Fig. 5:**
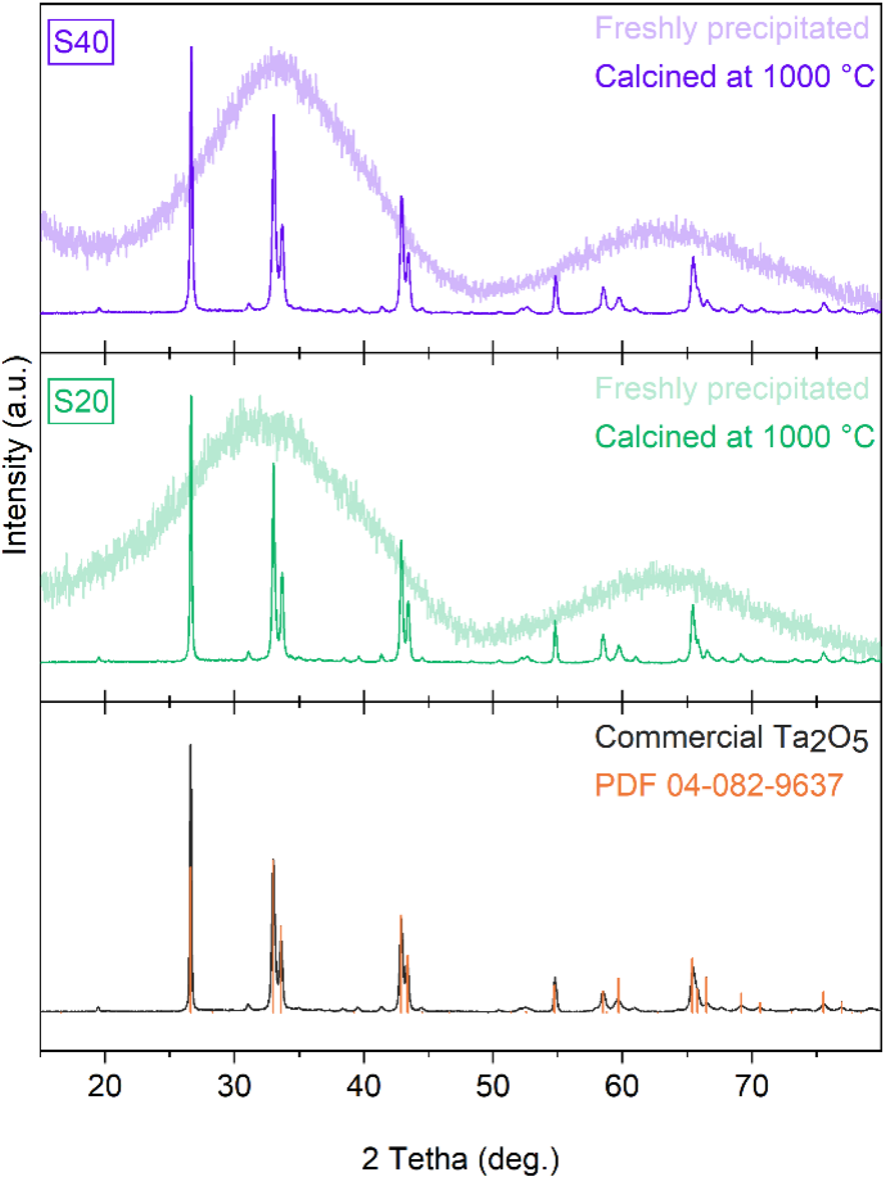
X-ray diffractograms of precipitates (freshly collected and calcined at 1000 °C) after acidification with HCl of the solution obtained by dissolving magnesium hexatantalate in EDTA at pH 11.

**Fig. 6:**
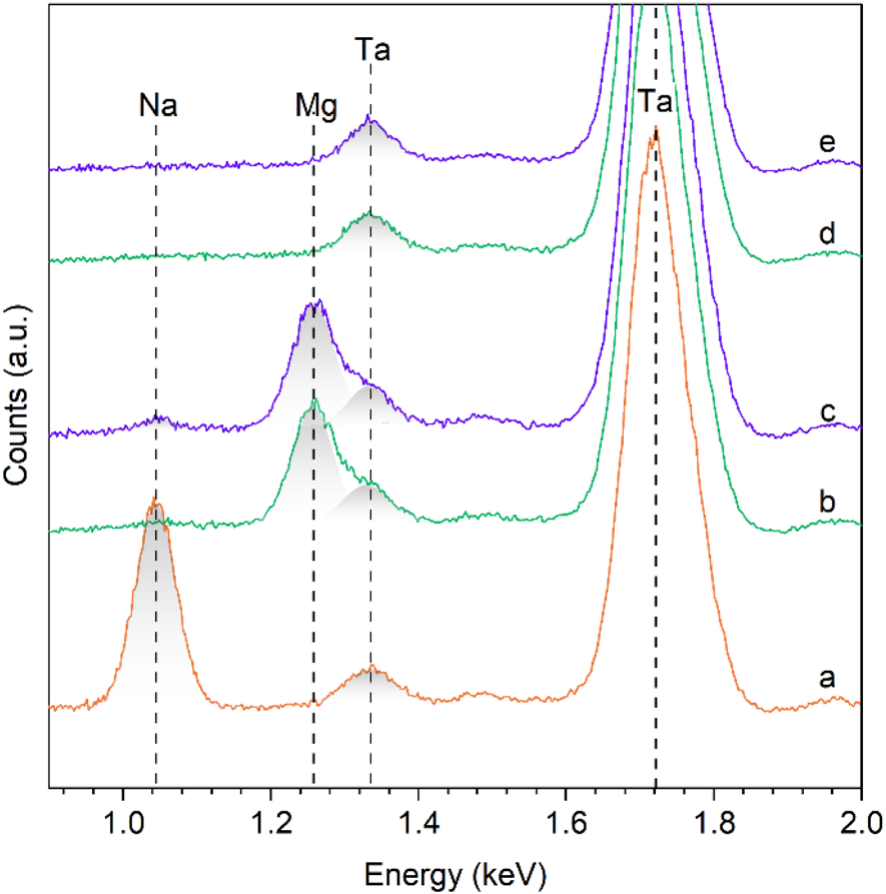
EDX spectra of (a) sodium hexatantalate and magnesium hexatantalate precipitated at (b) 20 °C and (c) 40 °C and of Ta_2_O_5_ after calcination of the sample precipitated at (d) 20 °C and (e) 40 °C.

From MgPOTa dissolution leachates using EDTA in excess of 10% compared to the required quantity, tantalum was finally re-precipitated as Ta_2_O_5_ by acidifying the solutions with HCl. Indeed, Ta_2_O_5_ begins to form at pH 9–10.^12^ It is important to note that the tantalum precipitation yield exceeds 99.9% since the concentration of tantalum remaining in the solution determined by ICP-OES is below the quantification limit (<0.07 ppm). The freshly obtained precipitates were then analyzed by PXRD. The diffraction patterns of the freshly precipitated solids (Fig. 5) are characteristic of an amorphous product, making phase identification impossible. After the calcination of the products at 1000 °C, the solids show diffractograms that can be attributed to pure crystalline Ta_2_O_5_. No peaks belonging to the other phases are observed. To evaluate the presence of impurities, such as magnesium and sodium, the calcined solids were subjected to EDX analysis (Fig. 6). The EDX spectra were then compared with those of NaPOTa and MgPOTa. The peaks of Mg and Na are absent in the Ta_2_O_5_ spectra. EDX results cannot exclude trace-level impurities below their detection limit, and further analysis is required for the given specifications of Ta purity depending on the final tantalum use. Moreover, no crystalline impurity phases were detected.

It should be emphasized that removing Na and Mg impurities was achieved after performing 3 washing steps with an acidic solution at pH 3, followed by 3 washing steps with ultrapure water. When the washing steps are conducted solely with ultrapure water, preliminary tests indicate the presence of sodium in the solid. The acidic solution helps lower the surface charge (in absolute value) of Ta_2_O_5_, whose point of zero charge is measured at 4.1,^23^ thereby diminishing the electrostatic attraction forces of the surface towards cations, such as Na^+^.

## Conclusions

This study presents an innovative approach to tantalum recovery from alkaline leachates, emphasizing the utilization of a series of processes that minimize environmental impact and do not require the use of hazardous substances. The proposed protocol is fluorine-free, and the precipitation of tantalum uses a harmless magnesium salt and a magnesium chelatant.

First, the alkaline polyoxotantalate leachates were mixed with a magnesium chloride solution to induce the precipitation of solid Mg_4_Ta_6_O_19_·*x*H_2_O compounds, without the effect of temperature between 20 °C and 40 °C. Two compounds corresponding to Mg_4_Ta_6_O_19_ · 16.1H_2_O and Mg_4_Ta_6_O_19_ · 24.3H_2_O are identified. The origin of the difference in the hydration of the precipitates depends on the drying protocols, which should be clarified in future studies. Starting with a ratio Mg/Ta_6_ ≥ 4 demonstrates the maximum tantalum precipitation yield (>99%). This quantitative tantalum precipitation is possible at alkaline pH, thus avoiding the preliminary neutralization of the alkaline leachate. The subsequent step consists of redissolving Mg–Ta precipitates using EDTA under alkaline conditions, still avoiding the neutralization of the soda. The molar amount of EDTA required is directly proportional to the quantity of Mg in the solid. Then, the precipitation of tantalum pentoxide is performed by neutralizing the solution since EDTA keeps magnesium in the solution, enabling its separation from tantalum. For a more sustainable approach, substituting EDTA with greener chelatants could be studied.^24^ Finally, an amorphous Ta_2_O_5_ phase free from Mg and Na impurities is obtained after washing several times with dilute acid and ultrapure water based on XRD and EDX results. Some complementary analyses with more sensitive methods could be performed to ensure that they meet the specifications of the intended application (electronics, superalloys, *etc.*). Based on these promising results, future efforts will focus on extending the process to real e-waste streams.

## Conflicts of interest

There are no conflicts to declare.

## Supplementary Material

RA-015-D5RA02721G-s001

## Data Availability

The data supporting this article have been included in the ESI.†
